# Supplementary material for: Distinct functions for beta and alpha bursts in gating of human working memory
Source: Nat Commun. 2024 Oct 17;15:8950. doi: 10.1038/s41467-024-53257-7 (PMC11486900; doi:10.1038/s41467-024-53257-7)
Supplement: Supplementary file 1 — Supplementary Information [file 41467_2024_53257_MOESM1_ESM.pdf]

Supplementary Information

DISTINCT FUNCTIONS FOR BETA AND ALPHA BURSTS IN GATING OF HUMAN WORKING MEMORY

Liljefors et al.

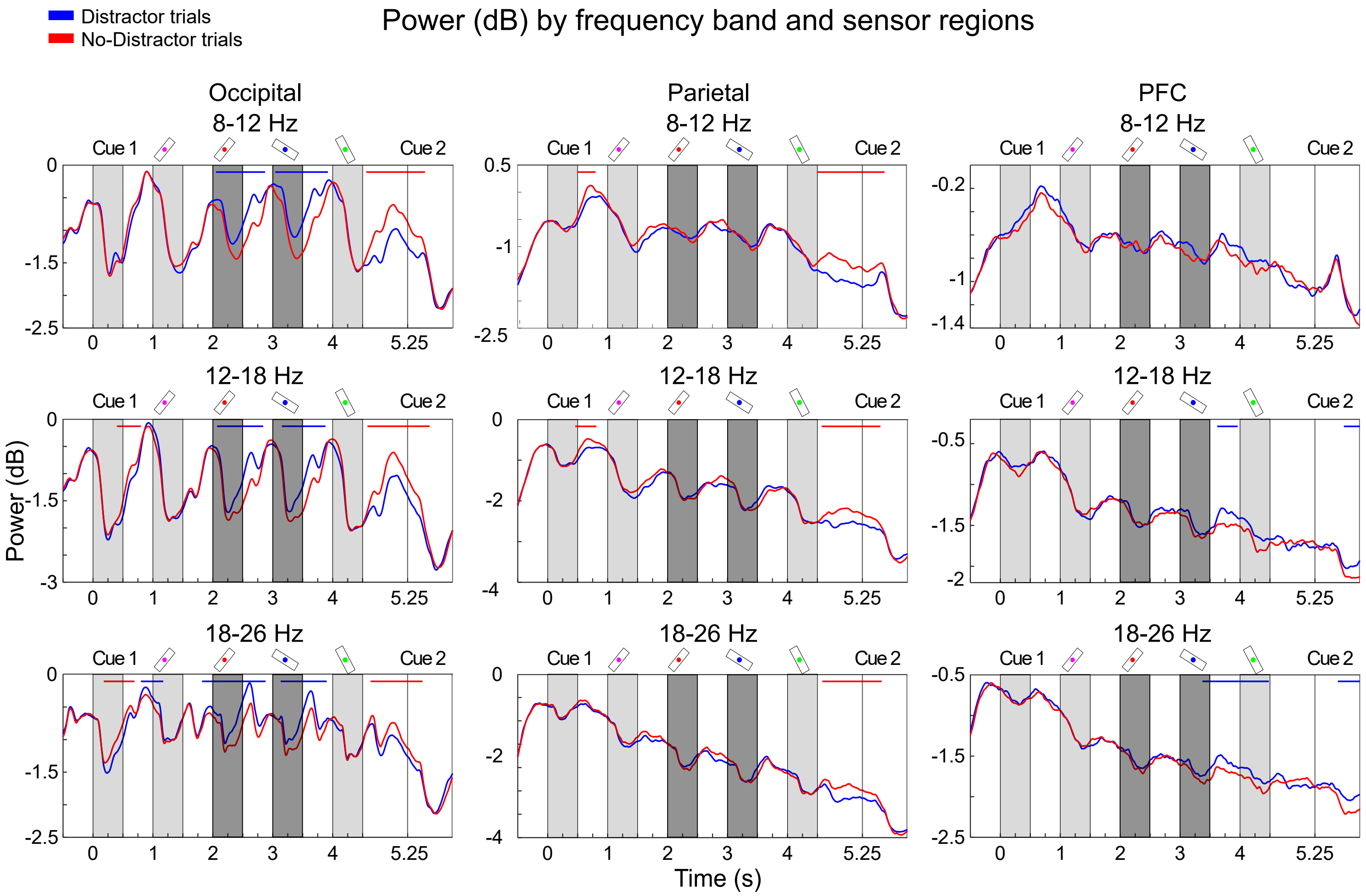

Figure S1. **Trial-averaged power by condition, band and region.** Trial-averaged spectral power baseline adjusted (dB, baseline period 0.25-0s before cue onset) for the three frequency bands and sensors of interest. Shaded areas represent stimuli presentation periods. Distractor presentation periods in Distractor trials (bar 2 and 3) are shown in dark (as opposed to light) grey areas. Red and blue bars denote periods of significant differences between conditions, using two-sided clusterbased permutation test at the p<0.001 level. Source data are provided as a source data file.

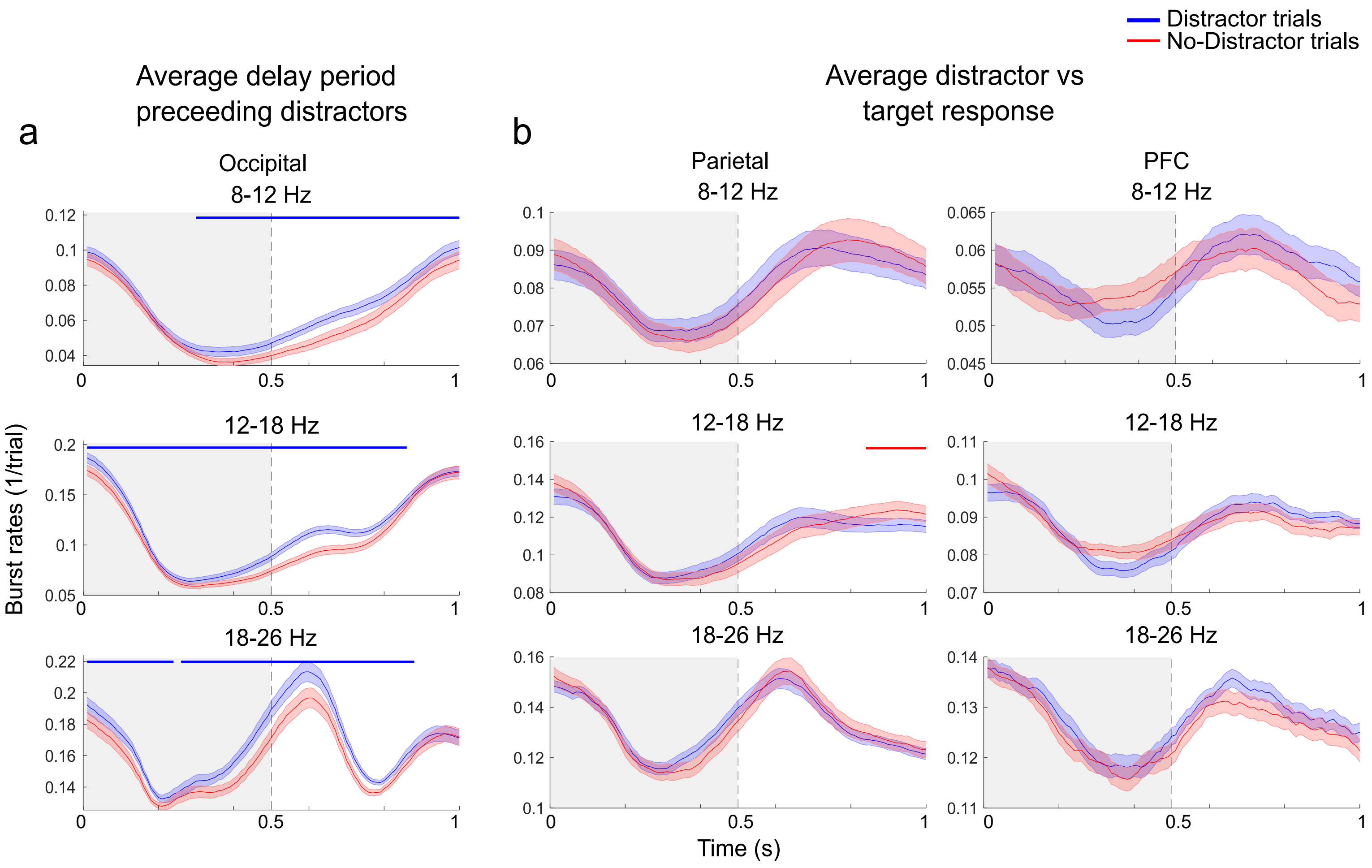

Figure S2. **Average burst rates by region and frequency. a** Occipital burst rates (shown in Figure 5a) but averaged over 1s periods starting from the onset of bars 1 and 2 for each frequency band and area. The white areas represent the delay periods preceding the presentation of distractors (blue, in Distractor trials) or targets (red, in No-Distractor trials) for bar 2 and 3 in the sequence. Blue bars denote when Distractor trial burst rates were significantly above No-Distractor trial burst rates, and red bars the opposite, using two-sided cluster-based permutation test at the p<0.001 level. The error bands indicate 1 SEM. **b** Burst rates (shown in Figure 5a) but averaged over 1s period starting from the onset of bars 2 and 3 (serving as distractors in Distractor trials) for each frequency band and sensor areas. The grey shaded area indicates the presentation period of bars 2 and 3. Blue bars denote when Distractor trial burst rates were significantly above No-Distractor trial burst rates, and red bars the opposite, using two-sided cluster-based permutation test at the p<0.001 level. The error bands indicate ±1 SEM. Source data are provided as a source data file.

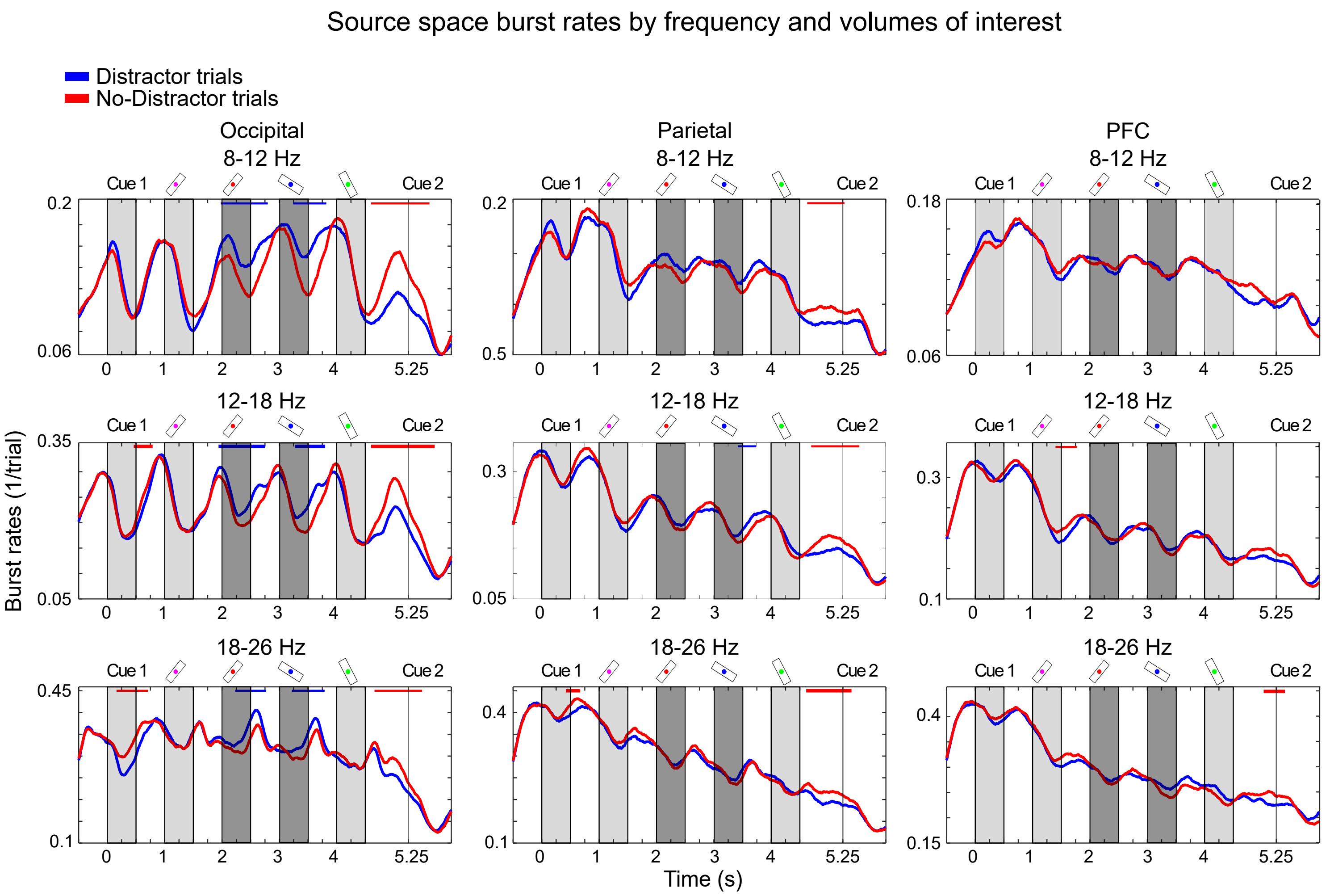

Figure S3. **Bursting by source space cortical region.** Burst rates for trial types and regions using source space computations (Methods). Burst rates per frequency band (rows) and region (columns) are shown. Burst rates for Distractor (blue) and No-Distractor (red) trials are plotted independently. Distractor presentation periods in Distractor trials (bar 2 and 3) are shown in dark (as opposed to light) grey areas. Blue bars denote when Distractor trial burst rates were significantly above No Distractor trial burst rates, and red bars the opposite, using two-sided cluster-based permutation test at the p<0.001 level. Source data are provided as a source data file.

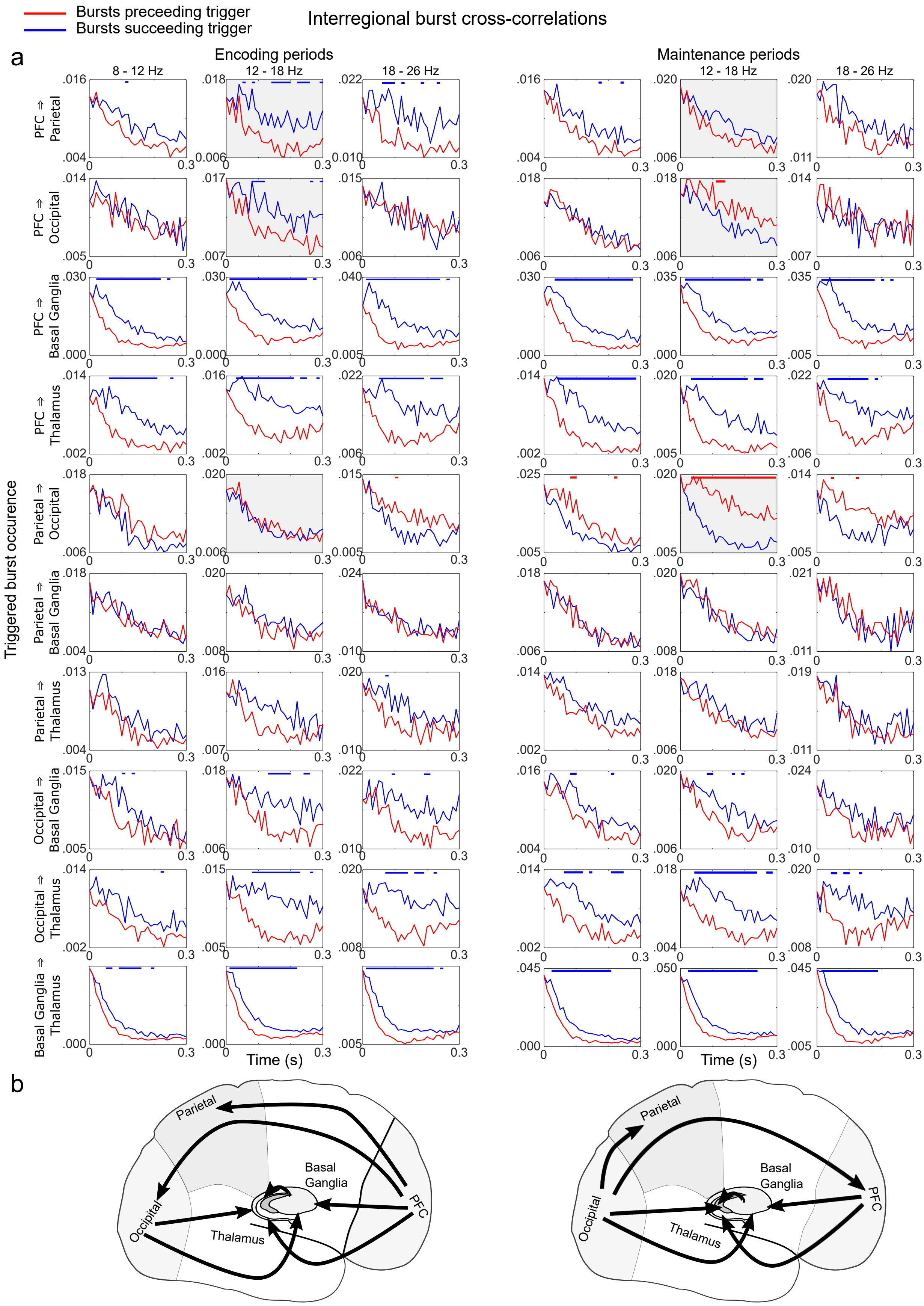

Figure S4. **Source space interregional burst cross-correlations. a** The x-axis at 0s marks the onset of a burst in the seed region (x →), while the y-axis represents the trial-averaged burst onset occurrence in the target region (→y) of interest. Red indicates bursts leading up to Time 0 ([-0.3,0] mirrored on the y-axis), and blue indicates bursts following the seed burst event. The three left columns correspond to the encoding period of the four stimuli (4 x 0.5s), and the right columns correspond to the subsequent maintenance periods (4 x 0.5s). Light grey area highlights differences between encoding and maintenance. See methods for AAL representations of source space volumes. Blue bars denote when bursts preceeding the triggering burst are significantly higher than bursts succeding the trigger, and red bars the opposite, using two-sided cluster-based permutation test at the $p < 0.01$ level. **b** Arrows illustrate the direction of burst-trigger interactions, indicating how low beta burst activity in one region precedes another. Source data are provided as a source data file.

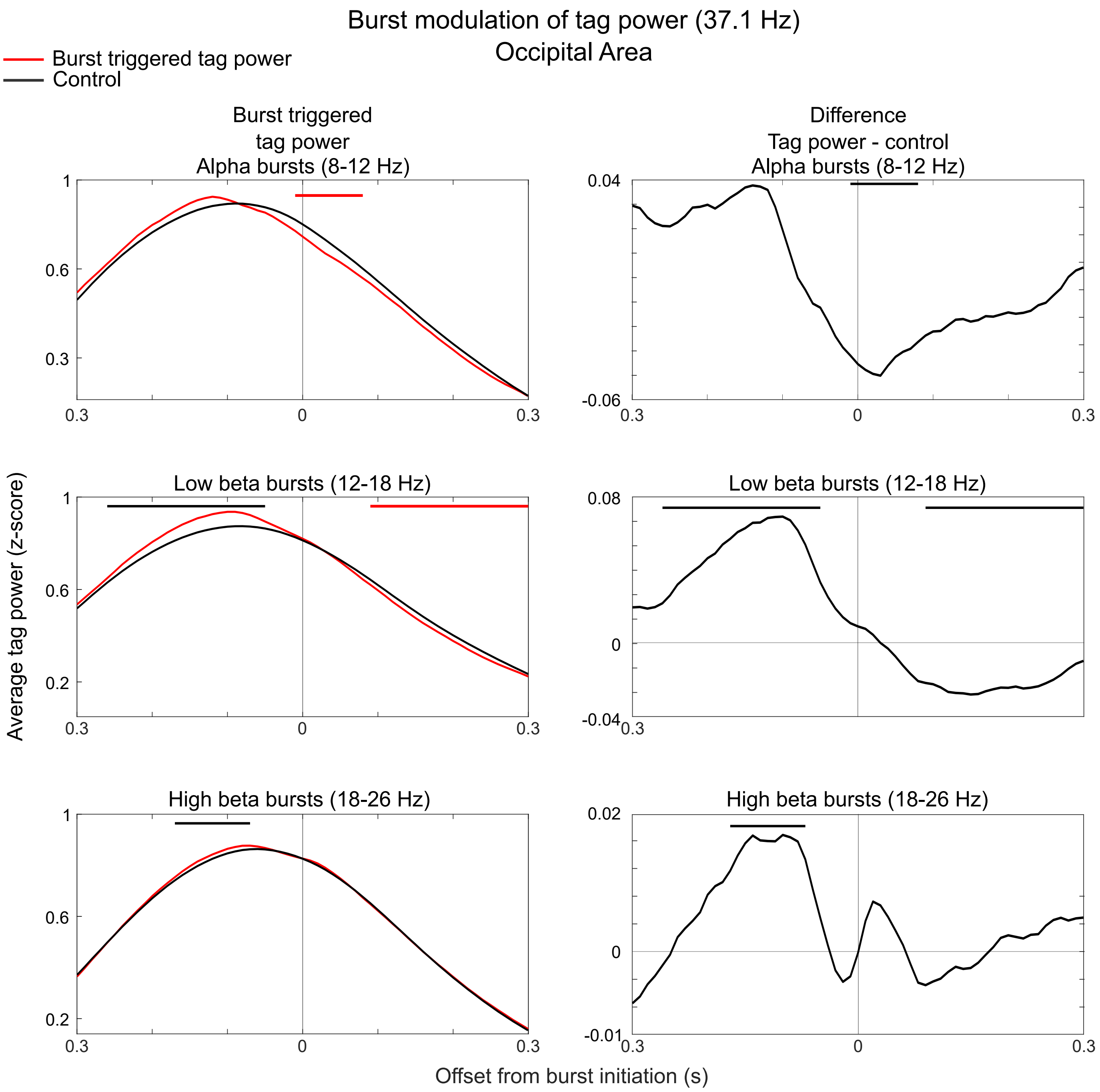

Figure S5. **Burst modulation of tag power for each frequency band.** Left column shows the burst triggered phase-locked power (37.1 ± 2 Hz, z-scored, see Methods) in red. Time 0 denotes the time of the detected burst onset. Displayed is also surrogate data, calculated by shuffling trial labels (within each condition and sensor independently) to estimate the power in the tagged frequencies drawn from the same distribution of times as the observed bursts (grey). Red bars denote periods in which power in the original data is lower than the surrogate data and grey bars indicate when power is higher in the original data than the surrogate data using two-sided permutation test at $p < 0.001$. The right column zooms in the difference between modulated tag power and surrogate data. The right column shows the difference between the actual data and the surrogate data. Source data are provided as a source data file.

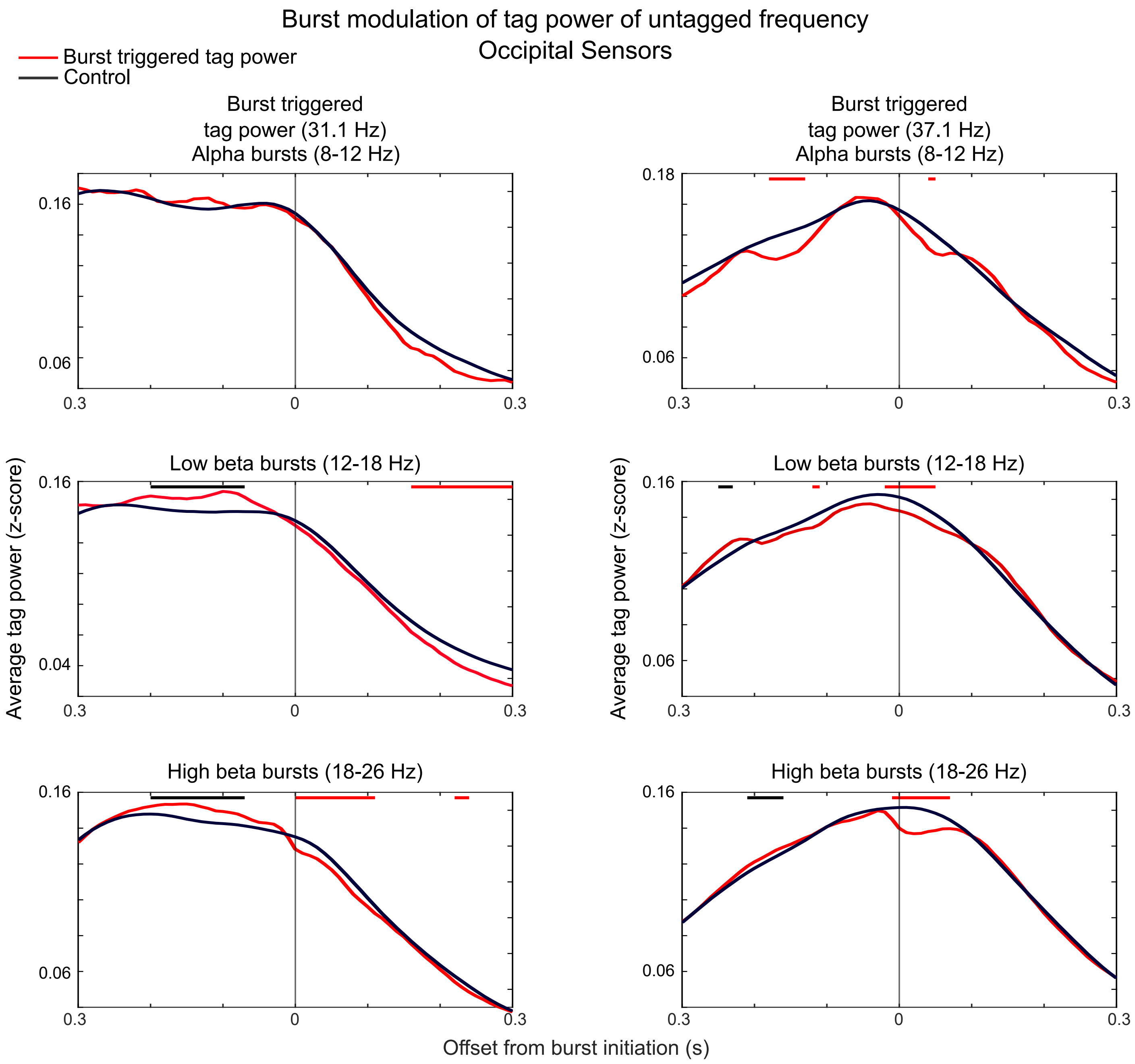

Figure S6. **Burst modulation of tag power of the untagged frequency, for the two frequency bands for occipital sensors.** Burst triggered average phase-locked power (z-scored, see Methods) averaged over the frequency bands (red). The left column shows the power at 31.1 Hz when the tagging frequency was 37.1 Hz, and the right column the power at 37.1 Hz when the tagging frequency was 31.1 Hz. Time 0 denotes the time of the detected burst onset. Displayed is also surrogate data, calculated by shuffling trial labels (within each condition and sensor independently) to estimate the power in the tagged frequencies drawn from the same distribution of times as the observed bursts (black). Red bars denote periods in which power in the original data is lower than the surrogate data and black bars indicate when power is higher in the original data than the surrogate data using twosided cluster-based permutation test at $p < 0.001$. Source data are provided as a source data file.

Sensor regions of interest

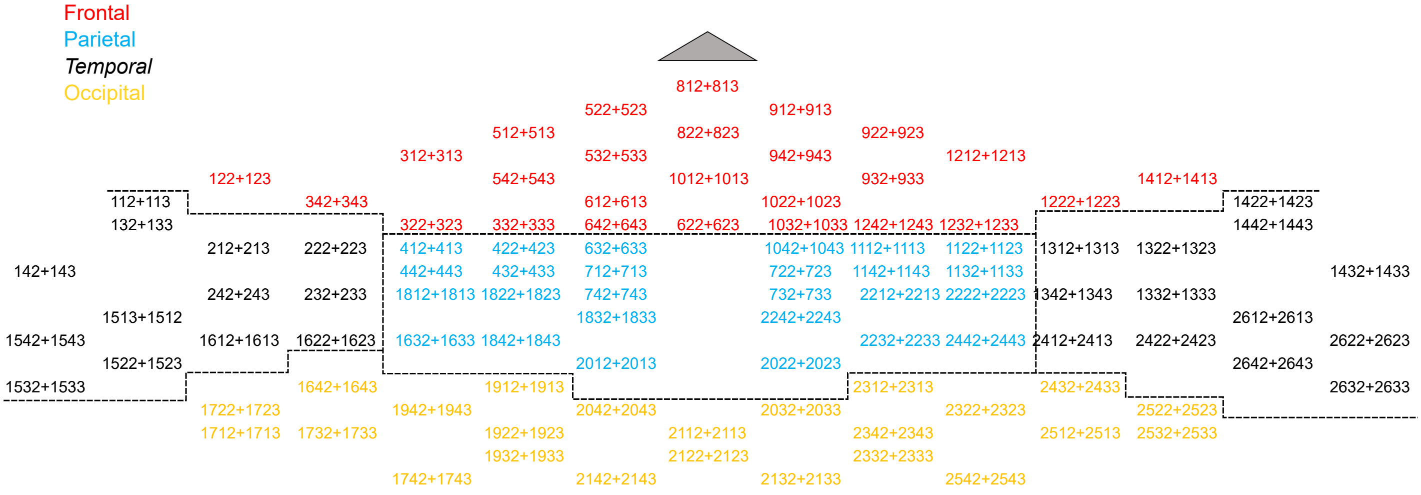

Figure S7. **MEG Synthetic sensor areas of interest and their underlying sensors.** Planar gradiometers were combined into synthetic gradiometers using Fieldtrip. Source data are provided as a source data file.

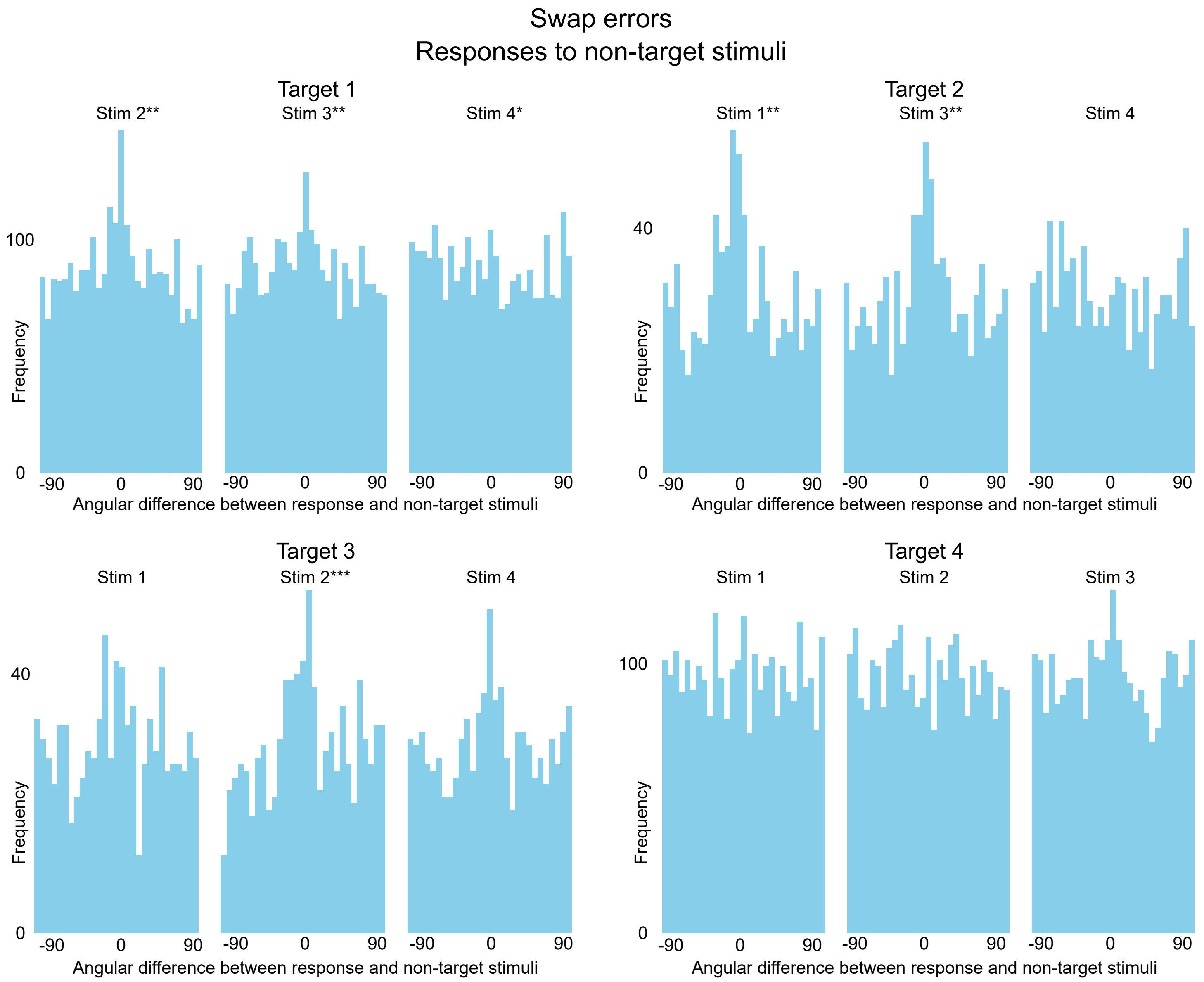

Figure S8. **Swap error analysis of responses to non-target stimuli.** Histogram of reported orientations, as angular distance between the bars that were not probed. Non-uniform distributions (tested with Kolmogorov-Smirnov tests and marked by * indicated evidence of swap errors where subjects reported the orientation of a non-probed bar. This occured for all targets except the last (helping explain why the accuracy was higher on the last bar). ** $p < 0.01$, *** $p < 0.001$. Source data are provided as a source data file.
