# Supplementary material for: Distinct functions for beta and alpha bursts in gating of human working memory
Source: Nat Commun. 2024 Oct 17;15:8950. doi: 10.1038/s41467-024-53257-7 (PMC11486900; doi:10.1038/s41467-024-53257-7)
Supplement: Supplementary file 3 — Reporting Summary [file 41467_2024_53257_MOESM3_ESM.pdf]

Corresponding author(s): Mikael Lundqvist

Last updated by author(s): Aug 12, 2024

Reporting Summary

Nature Portfolio wishes to improve the reproducibility of the work that we publish. This form provides structure for consistency and transparency in reporting. For further information on Nature Portfolio policies, see our Editorial Policies and the Editorial Policy Checklist.

Statistics

For all statistical analyses, confirm that the following items are present in the figure legend, table legend, main text, or Methods section.

n/a	Confirmed	
☐	☒	The exact sample size (*n*) for each experimental group/condition, given as a discrete number and unit of measurement
☐	☒	A statement on whether measurements were taken from distinct samples or whether the same sample was measured repeatedly
☐	☒	The statistical test(s) used AND whether they are one- or two-sided<br>*Only common tests should be described solely by name; describe more complex techniques in the Methods section.*
☐	☒	A description of all covariates tested
☐	☒	A description of any assumptions or corrections, such as tests of normality and adjustment for multiple comparisons
☐	☒	A full description of the statistical parameters including central tendency (e.g. means) or other basic estimates (e.g. regression coefficient) AND variation (e.g. standard deviation) or associated estimates of uncertainty (e.g. confidence intervals)
☐	☒	For null hypothesis testing, the test statistic (e.g. F, t, r) with confidence intervals, effect sizes, degrees of freedom and P value noted<br>*Give P values as exact values whenever suitable.*
☒	☐	For Bayesian analysis, information on the choice of priors and Markov chain Monte Carlo settings
☒	☐	For hierarchical and complex designs, identification of the appropriate level for tests and full reporting of outcomes
☐	☒	Estimates of effect sizes (e.g. Cohen's *d*, Pearson's *r*), indicating how they were calculated

Our web collection on statistics for biologists contains articles on many of the points above.

Software and code

Policy information about availability of computer code

Data collection	The experiment was presented using Presentation® software (Version 23.0, Neurobehavioural Systems, Inc., Berkeley, CA, www.neurobs.com), which also handled timing and event codes. The MEG scanner was an Elekta Neuroma TRIUX 306-channel, located inside a 2-layer mag-netically shielded room (www.natmeg.com). Cerebral data, EOG data, ECG data, event codes and responses were collected using Neuromag Acquisition software on a dedicated acquisition computer. T1 MRI Scans were collected at Stockholm University Brain Imaging center in a Siements Prisma 3 Tesla whole-body MRI.
Data analysis	Behavioural analysis was done using R version 2023.09.0 with the following packages: tidyr 1.3.0, dplyr 1.1.3, tidyverse 2.0.0, lme4 1.1.34, lmerTest 3.1.3, MASS 7.3.60, datawizard 0.9.1, performance 0.10.5, ggplot2 3.4.3 and circstats 0.2.6<br><br>All other analysis was done using Matlab R2022b, with toolboxes Fieldtrip 20221121, Parallel Computing Toolbox Version 7.7, RunLength Version 1.2.0.0

For manuscripts utilizing custom algorithms or software that are central to the research but not yet described in published literature, software must be made available to editors and reviewers. We strongly encourage code deposition in a community repository (e.g. GitHub). See the Nature Portfolio guidelines for submitting code & software for further information.

Data

Policy information about availability of data

All manuscripts must include a data availability statement. This statement should provide the following information, where applicable:

- Accession codes, unique identifiers, or web links for publicly available datasets
- A description of any restrictions on data availability
- For clinical datasets or third party data, please ensure that the statement adheres to our policy

Data Availability
The processed data, downsampled MEG data, MRI data and behavioural data used in this study are available in the OSF database under accession code CC BY [https://osf.io/gu25f/?view_only=6412a8bd665e4ef082385dbaa3d33026].

Code Availability
The code for this study is available for download from the Open Science Framework (OSF) homepage [https://osf.io/gu25f/?view_only=6412a8bd665e4ef082385dbaa3d33026].

Research involving human participants, their data, or biological material

Policy information about studies with human participants or human data. See also policy information about sex, gender (identity/presentation), and sexual orientation and race, ethnicity and racism.

Reporting on sex and gender	Participants were recruited with the intention of having a balance between the genders. No gender based analysis was relevant to our questions.
Reporting on race, ethnicity, or other socially relevant groupings	No social variables were used
Population characteristics	7 females and 10 males aged 21-41 years. Participants were screened for color blindness, and no cognitive impairments. 16 of the participants were students at Karolinska Institute, and one was a female employee of the same department as the primary and corresponding author however she was ignorant to the task prior to the experiment.
Recruitment	16 participants were students of the Psychology programme at Karolinska Institute. One participant was recruited externally and one was an employee of the department.
Ethics oversight	The Swedish Ethical Review Authority (Dnr 2021-00336) approved the study

Note that full information on the approval of the study protocol must also be provided in the manuscript.

Field-specific reporting

Please select the one below that is the best fit for your research. If you are not sure, read the appropriate sections before making your selection.

☒ Life sciences ☐ Behavioural & social sciences ☐ Ecological, evolutionary & environmental sciences

For a reference copy of the document with all sections, see nature.com/documents/nr-reporting-summary-flat.pdf

Life sciences study design

All studies must disclose on these points even when the disclosure is negative.

Sample size	Sample size was predetermined based on past experience with EEG and intracranial experiments
Data exclusions	One male subject did not perform the experiment due to magnetic interference, and another male subject was excluded due to excessive movement resulting in poor data quality.
Replication	The experiment was internally replicated within participants, with each participant completing 400 trials under two different conditions. This setup allowed for the assessment of the consistency of the effect within individuals across multiple trials. Additionally, the study was replicated across 17 participants to determine the generalizability of the findings across different individuals. The observed effects were consistent both within participants across trials and between participants across conditions.
Randomization	Pseudo-randomization was used to generate the order of the trials. 200 trials of each conditions, where pseudorandomized to appear random, but with no more than 3 consecutive trials of the same condition
Blinding	Blinding was not used, each subject was made aware of the condition of each trial from a cue presented at initiation of each trial

Reporting for specific materials, systems and methods

We require information from authors about some types of materials, experimental systems and methods used in many studies. Here, indicate whether each material, system or method listed is relevant to your study. If you are not sure if a list item applies to your research, read the appropriate section before selecting a response.

Materials & experimental systems

n/a	Involved in the study
☒	☐ Antibodies
☒	☐ Eukaryotic cell lines
☒	☐ Palaeontology and archaeology
☒	☐ Animals and other organisms
☒	☐ Clinical data
☒	☐ Dual use research of concern
☒	☐ Plants

Methods

n/a	Involved in the study
☒	☐ ChIP-seq
☒	☐ Flow cytometry
☒	☐ MRI-based neuroimaging

Plants

Seed stocks

Report on the source of all seed stocks or other plant material used. If applicable, state the seed stock centre and catalogue number. If plant specimens were collected from the field, describe the collection location, date and sampling procedures.

Novel plant genotypes

Describe the methods by which all novel plant genotypes were produced. This includes those generated by transgenic approaches, gene editing, chemical/radiation-based mutagenesis and hybridization. For transgenic lines, describe the transformation method, the number of independent lines analyzed and the generation upon which experiments were performed. For gene-edited lines, describe the editor used, the endogenous sequence targeted for editing, the targeting guide RNA sequence (if applicable) and how the editor was applied.

Authentication

Describe any authentication procedures for each seed stock used or novel genotype generated. Describe any experiments used to assess the effect of a mutation and, where applicable, how potential secondary effects (e.g. second site T-DNA insertions, mosiacism, off-target gene editing) were examined.
